# Transfer of *Meloidogyne incognita* Resistance Using Marker-assisted Selection in Sorghum

**DOI:** 10.21307/jofnem-2021-087

**Published:** 2021-11-11

**Authors:** Richard F. Davis, Karen R. Harris-Shultz, Joseph E. Knoll, Hongliang Wang

**Affiliations:** 1 USDA-ARS, Crop Genetics and Breeding Research Unit, P.O. Box 748, Tifton, GA 31793; 2 USDA-ARS, Hard Winter Wheat Genetics Research, 4007 Throckmorton Hall, Manhattan, KS 66506

**Keywords:** Breeding, Markers, Nematode resistance, QTL, Sorghum bicolor, *Southern root-knot nematode*

## Abstract

*Meloidogyne incognita* is a wide-spread and damaging pathogen of many important crops in the southern United States, and most sorghum genotypes allow significant levels of reproduction by the nematode. A series of greenhouse evaluations were conducted to determine whether a quantitative trait locus (QTL) that imparts a high level of resistance to *Meloidogyne incognita* in sorghum can effectively be transferred into diverse sorghum genotypes using marker assisted selection. Using marker-assisted selection, the resistance QTL, QTL-Sb.RKN.3.1, from ‘Honey Drip’ sorghum was crossed into five different sorghum backgrounds that included forage, sweet, and grain sorghum until the BC_1_F_6_ generation. Repeated greenhouse experiments documented that the recurrent parent genotypes were all susceptible to *M. incognita* and statistically similar to each other. In contrast, the BC_1_F_6_ genotypes were all highly resistant and similar to each other and similar to the resistant standard, ‘Honey Drip’. These results suggest that this resistance QTL could be introgressed using marker assisted selection into many sorghum genotypes and confer a high level of resistance to *M. incognita*. Thus, this QTL and its associated markers will be useful for sorghum breeding programs to incorporate *M. incognita* resistance into their sorghum lines.

Sorghum (*Sorghum bicolor*) is well adapted to semi-arid tropical areas and is an important food source for humans and livestock worldwide (Babatola and Idowu, 1990). Several types of sorghum have been selected within *S. bicolor* to meet different needs: grain sorghum is solely for grain production, forage sorghum is for forage and silage production, and sweet sorghum is for syrup and sugar production. An overwhelming majority of the sorghum grown in the United States is grain sorghum. In the United States in 2018, sorghum was planted on approximately 6 million acres, largely in the Great Plains (USDA-NASS 2019), and it is often grown as a rotation crop with cotton (*Gossypium hirsutum*), corn (*Zea mays*), or soybeans (*Glycine max*) (Xavier-Mis et al., 2017). Because of its drought tolerance and relatively low input requirements, sorghum could be more widely used as a cost-effective rotation crop in the southeastern US.

*Meloidogyne incognita* is the most commonly occurring species of root-knot nematode in warm temperate to tropical agroecosystems around the world, and it causes significant economic damage to many crops in the US (Sasser and Carter, 1985). Many genotypes of sorghum are good hosts for *M. incognita* (Xavier-Mis et al., 2017), and *M. incognita* is the most important *Meloidogyne* species on sorghum (McGawley and Overstreet, 1998). Sorghum appears to suffer relatively little damage from root-knot nematodes even on genotypes that are good hosts with overall losses estimated at 1.5% in the US (Koenning et al., 1999), however, significant yield reductions caused by *M. incognita* have been reported in selected fields (Orr, 1967; Thomas and Murray, 1987). An increase in days to flowering and tillering as well as yield losses with increasing levels of *M. incognita* inoculum was reported for pot studies using five sorghum cultivars (Babatola and Idowu, 1990). The increase of *M. incognita* levels on susceptible sorghum would pose a significant risk for subsequently planted susceptible crops such as cotton. Although farmers are unlikely to apply a nematicide to a sorghum crop because of the added expense, they may be willing to plant a resistant genotype to reduce damage from *M. incognita* in future crops. Currently, the limited availability of resistant cultivars restricts the use of sorghum as a rotation crop to suppress *M. incognita* (Xavier-Mis et al., 2017).

The host status of sorghum hybrids for *M. incognita* has been reported as ranging from highly resistant to highly susceptible. Fortnum and Currin (1988) reported that a selection of 10 hybrids were all poor hosts for *M. incognita* and suppressed nematode levels in the field in South Carolina. Studies in Florida reported one sorghum hybrid as a poor host (McSorely and Gallaher, 1991) and another as a good host for *M. incognita* (McSorely and Gallaher, 1992), so the authors concluded that hybrid selection was crucial for managing *M. incognita* (McSorely and Gallaher, 1992). Controlled reproduction studies have also found mixed results for the susceptibility of sorghum, but most hybrids were found to range from moderately susceptible to very susceptible with only a few being resistant (De Brida et al., 2017; Hurd and Faske, 2017; Xavier-Mis et al., 2017).

In plants, although many quantitative trait loci (QTL) have been identified and associated with various traits, few are pursued beyond the initial identification (Collard et al., 2005). Bernardo (2008) notes that “the vast majority of the favorable alleles at the identified QTL reside in journal shelves rather than in cultivars.” Resistant cultivars provide cost effective and efficient nematode control (Starr et al., 2002). A QTL (QTL-Sb.RKN.3.1) that imparts a high level of resistance to *M. incognita* in sorghum was identified in the sweet sorghum cultivar ‘Honey Drip’, and microsatellite markers were identified in the QTL region (Harris-Shultz et al., 2015a). This QTL on Chr. 3 contributed 70% of the phenotypic variance for the number of *M. incognita* eggs per g of root. The goal of this project was to verify that the *M. incognita* resistance QTL (QTL-Sb.RKN.3.1) and its associated markers could be used effectively for marker-assisted selection (MAS) to incorporate into susceptible sorghum genotypes a level of resistance to *M. incognita* equal to that of the resistant parent.

## Materials and methods

### Development of BC_1_F_6_ lines

The recurrent, susceptible parents used in this study were Collier, Dale, Entry 22, GT-IR7, and Top 76-6 (Table 1). The recurrent parents included sweet, forage, and grain type sorghum and were all susceptible to *M. incognita* (pers. obs.), whereas the donor parent, ‘Honey Drip’, was highly resistant (Harris-Shultz et al., 2015a). Collier, Dale, and Top 76-6 are commonly used lines for sweet sorghum production, Entry 22 is an experimental forage line from the University of Florida, and GT-IR7 is a grain type with resistance to leaf feeding by fall armyworm (*Spodoptera frugiperda*) and sorghum midge (*Contarinia sorghicola*) (Table 1, Widstrom, 1998). The BC_1_F_6_ lines are referred to herein by adding “-BC1F6” to the recurrent parent name (Collier-BC1F6, Entry 22-BC1F6, Dale-BC1F6, Top 76-6-BC1F6, and GT-IR7-BC1F6). To create the BC_1_F_6_ lines for testing, sorghum was grown in two-gallon pots in a greenhouse in soil containing a 1:1:1 mixture of masonry sand (Double A Concrete, Tifton, Georgia), peat moss (PremierTech Horticulture, Quakertown, Pennsylvania), and coarse perlite, plus 23 g/L dolomitic lime (Harris-Shultz et al., 2015b). To prevent self-pollination, heads of ‘Honey Drip’ were hand emasculated using an angled point teasing needle (ThermoFisher Scientific, Waltham, MA) to remove the immature anthers from the florets. On the morning after emasculation, and for the following two mornings, crosses were made by transferring pollen using paper bags from five susceptible sorghum lines to five heads of the resistant parent, ‘Honey Drip’ (one head per recurrent parent), to produce seed for F_1_ plants. The seeds were allowed to mature (approximately 45 days after pollination), and the heads were harvested and dried.

**Table 1.** Recurrent sorghum parents used for marker-assisted selection of the *M. incognita* resistance QTL, QTL-Sb.RKN.3.1, from ‘Honey Drip’ (PI 641821).

Name	PI number	Reference/source	Sorghum type
Collier	PI 641862	Maunder (2000)	sweet
Dale	PI 651495	Broadhead and Coleman (1973)	sweet
Entry 22	–	University of Florida	forage
GT-IR7	PI 602445	Widstrom (1998)	grain
Top 76-6	PI 583832	Day et al. (1995)	sweet

To identify F_1_ plants, the potential F_1_ seed was grown (20 seeds per cross) and leaf tissue was harvested for DNA extraction approximately 30 days after emergence. Tissue was cut into approximately 0.5 cm pieces and placed into 2 mL microcentifuge tubes containing four Zn-plated BBs (Daisy Outdoor Products, Rogers, AR). The tubes containing the tissue and beads were placed into liquid N_2_, and the contents were ground on a vortex mixer until the tissue formed a fine powder. The tubes were repeatedly placed back into liquid N_2_ to prevent the tissue from thawing. DNA was then extracted using a GeneJET Plant Genomic DNA Purification kit (ThermoFisher Scientific). True F_1_ plants were identified by genotyping using two to four sorghum microsatellite markers, which included TRKN1, TRKN3, TRKN4, TRKN5, RKNP194, RKNP259, RKNP342, RKNP402, and RKNP529 (Table 2, Supplementary Table 1).

**Table 2.** Sorghum primer sequences of microsatellite markers used for confirming sorghum crosses in the *M. incognita* resistance quantitative trait locus region.

Marker	Forward sequence	Reverse sequence	Repeat motif	Expected amplicon (bp)a
RKNP17	GCAGTTTTTCAAGGAACGTG	GAGGAATGGGTGATGAAACAA	(TTA)106	422
RKNP135	GTTTCGTTTCAATCGGCTTC	GCGCCCCATCATATGTCTT	(AAG)21	197
RKNP194	TCATACTACCACAGCCGCTAGA	TGGTGTAGATGTGTGTGATTCAA	(AT)40	236
RKNP259	AGCTCTTCAGGCACATCGTT	TCTCCTTCCCACCCTGTATG	(GA)48	243
RKNP342	TTCCAACAGGCAAACAACAG	TCATGGCCTGTGTATCAAGC	(CT)25	205
RKNP402	TCAGCAAGATGGTTGGTTGA	ACGAGGCCGTTGAGATTATG	(TTA)22	213
RKNP465	TGACTGAGAGGGTCTACCTAACG	CAACCGGAAGTACGCTGATT	(AT)19	247
RKNP529	GCGAAATGGAGAAGAACAGG	CGTCATCAGCTTCCAGGAGT	(GA)25	199
RKNP638	CCACACCGGTTTCCTGTTAT	TAATAAGCCCCGCATGAAGA	(ATA)21, (TAT)6, (TAA)25	251
RKNP709	GCAAGCTGAAGTGGCCTAGT	CTACTCCCTCCGTCCCAAAT	(CT)9, (TA)18	324
RKNP821	CTCGGCAGCACCAAATAAAA	TCTCAACCGATGATTGTCCA	(TA)51	199
TRKN1	TGTACACTGCATGCCAACCT	GCCTCGTCTGGTTCATTGTT	(AT)14	246
TRKN3	GAAGAATTGCTCCAGGAACG	AAGCAGTATCCGGGGAAGAT	(TA)10	271
TRKN4	CGTAAATGGAGGTGGCTACA	CCCGGTTGGTACAACATAGA	(AT)29	232
TRKN5	ACTGTTATGTCGGCTGGTCA	AGTGTTACTGCCTGGCCAAA	(TC)11	196

Note: ^a^Expected amplicon size was obtained from the genomic sequence of sorghum genotype BTx623.

**Table S1.** Genotyping information for the creation of sorghum BC_1_F_6_ lines.

Date of PCR	Sorghum seedling(s) with confirmed cross	Microsatelite markers used
7/17/2015	Honey Drip x Collier	TRKN1, TRKN3, TRKN4
8/4/2015	Honey Drip x GT-IR7	TRKN1, TRKN3, TRKN4, TRKN5
8/4/2015	Honey Drip x Entry 22	TRKN1, TRKN3, TRKN4, TRKN5
8/4/2015	Honey Drip x Entry 22	TRKN1, TRKN3, TRKN4, TRKN5
8/25/2015	Honey Drip x Dale	TRKN1, TRKN3, TRKN4, TRKN5
11/30/2015	Honey Drip x Top 76-6	RKNP402, RKNP342
11/30/2015	Entry 22 x (Honey Drip x Entry 22)	RKNP402, RKNP342
12/16/2015	Honey Drip x Top 76-6	RKNP194, RKNP259, RKNP402, RKNP529
1/26/2016	Dale x (Honey Drip x Dale)	RKNP194, RKNP259, RKNP465, RKNP529
2/19/2016	GT-IR7 x (Honey Drip x GT-IR7)	TRKN4, TRKN3, RKNP529, RKNP638
2/29/2016	Collier x (Honey Drip x Collier)	RKNP529, RKNP638
3/9/2016	GT-IR7 x (Honey Drip x GT-IR7)	RKNP529, TRKN3, RKNP638
3/14/2016	GT-IR7 x (Honey Drip x GT-IR7)	RKNP638, RKNP194, RKNP465
3/15/2016	GT-IR7 x (Honey Drip x GT-IR7)	RKNP638, RKNP194, RKNP465
3/28/2016	Entry 22 x (Honey Drip x Entry 22) F_2_ homozygous	RKNP342, RKNP402, RKNP529
3/28/2016	Collier x (Honey Drip x Collier)	RKNP342, RKNP402, RKNP529
4/20/2016	Top 76-6 x (Honey Drip x Top 76-6)	RKNP342, RKNP402, RKNP529
4/20/2016	Entry 22 x (Honey Drip x Entry 22) F_2_ homozygous	RKNP342, RKNP402, RKNP529
4/21/2016	Entry 22 x (Honey Drip x Entry 22) F_2_ homozygous	RKNP342, RKNP402, RKNP529
5/6/2016	Dale x (Honey Drip x Dale) F_2_ homozygous	RKNP342, RKNP402, RKNP529
5/11/2016	Dale x (Honey Drip x Dale) F_2_ homozygous	RKNP342, RKNP402, RKNP529
6/1/2016	Dale x (Honey Drip x Dale) F_2_ homozygous	RKNP342, RKNP402, RKNP529
6/8/2016	Dale x (Honey Drip x Dale) F_2_ homozygous	RKNP342, RKNP402, RKNP529
6/9/2016	GT-IR7 x (Honey Drip x GT-IR7) F_2_ homozygous	RKNP342, RKNP402, RKNP529
6/22/2016	Collier x (Honey Drip x Collier) F_2_ homozygous	RKNP342, RKNP402, RKNP529
8/30/2016	Top76-6 x (Honey Drip x Top 76-6) F_2_ homozygous	RKNP529, RKNP638, RKNP709, RKNP821
6/15/2017	Genotyping of BC_1_F_6_ lines to determine the size of the Honey Drip crossover in the RKN region	RKNP17, RKNP135, RKNP342, RKNP402, RKNP529

Note: Many seedlings were created for each cross. At the F_2_ homozygous stage (where the F_2_ plant is homozygous for Honey Drip in the *Meloidogyne incognita* resistance QTL region), a single seedling was selected from each backcross and advanced to the F_6_ stage.

The confirmed F_1_ plants were then grown and used as the pollen parent for backcrossing to each recurrent parent listed in Table 1. As described above, the resulting seed (BC_1_F_1_) was grown, DNA was extracted, and two to four microsatellites in the *M. incognita* resistance gene region (depending on the polymorphism of the parents), RKNP194, RKNP259, RKNP342, RKNP402, RKNP465, RKNP529, RKNP638, TRKN3, and TRKN4 (Table 2, Supplementary Table 1), were used to identify those plants that contain an allele from ‘Honey Drip’ in the *M. incognita* resistance QTL region. Those plants that were confirmed as BC_1_F_1_ and carrying a ‘Honey Drip’ allele for QTL-Sb.RKN.3.1 were self-pollinated to generate BC_1_F_2_ seed. Plants were genotyped at this stage using three to four markers that included RKNP342, RKNP402, RKNP529, RKNP638, RKNP709, and RKNP821 (Table 2, Supplementary Table 1) to identify plants that were homozygous for the ‘Honey Drip’ allele in the QTL-Sb.RKN.3.1 region. Those plants that were homozygous were advanced to the BC_1_F_6_ stage through repeated generations of self-pollination. The size of the introgression from Honey Drip in each line was determined in the BC_1_F_6_ generation by using five markers, RKNP17, RKNP135, RKNP342, RKNP402, and RKNP529 (Table 3, Supplementary Table 1) that span the root-knot nematode resistance gene region.

**Table 3.** Presence of the introgression from ‘Honey Drip’ in the QTL-Sb.RKN.3.1 region of each backcross (BC_1_F_6_) line.

	Allele Sizes (bp)				
	RKNP17^a^	RKNP135	RKNP342	RKNP402	RKNP529
NC_012872 (bp)b	(52,744,750)	(53,190,542)	(53,974,058)	(54,199,591)	(54,678,311)
*Genotype*					
Honey Drip	***120, 120***	***153, 153***	***240, 240***	***173, 173***	***222, 222***
Entry 22	198, 198	210, 210	222, 222	204, 204	185, 185
Entry 22-BC1F6	***120, 120***	***153, 153***	***240, 240***	***173, 173***	***222, 222***
Collier	198, 198	200, 200	220, 220	253, 253	116, 116
Collier-BC1F6	***120, 120***	***153, 153***	***240, 240***	***173, 173***	***222, 222***
GT-IR7	198, 245	210, 210	220, 220	265, 265	116, 232
GT-IR7-BC1F6	***120, 120***	***153, 153***	***240, 240***	***173, 173***	***222***, 232
Dale	198, 198	206, 206	222, 222	234, 234	185, 185
Dale-BC1F6	***120, 120***	***153, 153***	***240, 240***	***173, 173***	***222, 222***
Topper	198, 198	202, 202	220, 220	255, 255	116, 232
Top 76-6-BC1F6	***120, 120***	***153, 153***	***240, 240***	***173, 173***	***222***, 232

Notes: ^a^RKNP17, RKNP135, RKNP342, RKNP402, and RKNP529 are microsatellite markers in the QTL-Sb.RKN.3.1 region.

b The base pair position is the start site of the forward primer on NC_012872, the sorghum chromosome 3 genomic sequence. Allele sizes in bold italics indicate this allele is from ‘Honey Drip’.

### Evaluation of resistance

Reproduction of *M. incognita* on the genotypes in this study was documented in two greenhouse trials with 6 replications in a randomized complete block design for each trial. The cultivar Collier was used as a susceptible standard, and Honey Drip was used as a resistant standard. Two seeds were planted into 15-cm-diameter pots containing steam-pasteurized field soil (Tifton Loamy Sand), and seedlings were thinned to one plant per pot prior to inoculation. Inoculum was collected from eggplant roots (*Solanum melongena* L.) by agitating roots in 0.5% NaOCl solution for two minutes (Hussey and Barker, 1973) approximately 1 hour before inoculation. Inoculum of 8,000 *M. incognita* eggs/pot (approximately 600 eggs/150 cm^3^ soil) was distributed into two holes (approximately 2.5 cm deep) and covered with soil. Pots were watered immediately following inoculation.

Nematode eggs were extracted from the entire root system of each plant 56 days after inoculation. Roots were washed free of soil, weighed, cut into 5-cm pieces, and agitated in a 1.0% NaOCl solution in a 1-liter flask for four minutes (Hussey and Barker, 1973). Eggs were collected and rinsed with tap water on nested 150- over 25-µm-pore sieves. Egg counts and eggs/g root were subjected to a log_10_ transformation to equalize the error variances prior to statistical analysis. Data from the two trials were pooled for a combined analysis. Data were analyzed by mixed model analysis using PROC GLIMMIX in SAS with replication as a random effect and genotype and trial as fixed effects. Statistical differences among means were identified using the LSMEANS statement with the DIFF option.

## Results

**Figure 1:**
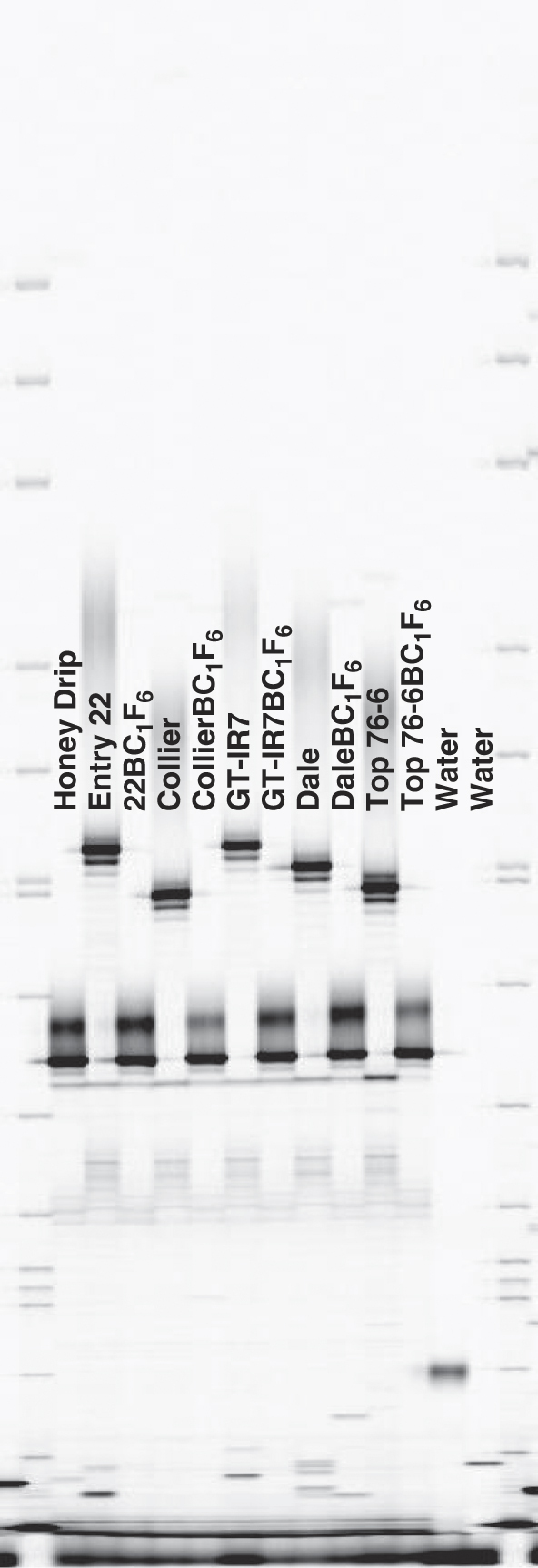
Polyacrylamide gel image of microsatellite marker RKNP135 from the sorghum donor line ‘Honey Drip’, the recurrent parents ‘Collier’, GT-IR7, ‘Dale’, and ‘Top 76-6’, and the BC_1_F_6_ progeny lines. A 50-350 bp sizing standard (LI-COR Biosciences, Lincoln, NE) was loaded in the first and last lanes.

Genotyping of each BC_1_F_6_ line with microsatellite markers in the QTL-Sb.RKN.3.1 region confirmed that each line contained DNA from Honey Drip in this region (Table 3, Fig. 1). GT-IR7-BC1F6 and Top 76-6-BC1F6 were both heterozygous for QTL-Sb.RKN.3.1 marker RKNP529, which is at 54,678,311 bp on the sorghum chromosome 3 genomic sequence (Table 3).

The fresh weights of the sorghum root systems differed among genotypes in both trials (Table 4). However, there was a significant statistical Trial × Genotype interaction (*P* = 0.0190), so each trial was analyzed separately; the interaction appears to have been due to greater differences in root weight in Trial 2 rather than to inconsistent relative performance of the genotypes. In both trials, Entry 22 had the lowest numerical root weight and Top 76-6 had the greatest numerical root weight (Table 4). In Trial 1, the root weight of each BC_1_F_6_ genotype was statistically similar to its recurrent parent (Table 4). However, in Trial 2, Top 76-6-BC1F6 had lower root weight than Top 76-6, whereas the other BC_1_F_6_ genotypes did not differ from their recurrent parent (Table 4).

**Table 4.** Fresh root weights of parental sorghum genotypes and their BC_1_F_6_ progeny.

	Trial 1		Trial 2	
Genotype	Root weight (g)a		Root weight (g)a	
Top 76-6	63.3	A^b^	97.8	A^b^
Top 76-6-BC1F6	56.6	AB	69.2	B
Honey Drip	43.9	BC	52.6	BCD
Dale-BC1F6	41.4	BCD	53.7	BC
Entry 22-BC1F6	41.1	CD	43.3	CDE
Collier	40.9	CD	32.9	DE
Collier-BC1F6	40.1	CD	31.5	E
GT-IR7	38.5	CD	27.9	E
GT-IR7-BC1F6	38.3	CD	30.6	E
Dale	37.1	CD	39.5	CDE
Entry 22	26.6	D	24.0	E

Notes: ^a^Root weights are from plants 8 weeks after infection with *M. incognita*.

b Means within a column followed by the same letter are not statistically different (*P* ≤ 0.05).

There was no Trial × Genotype interaction for the total number of nematode eggs produced, therefore, the trials were combined for a pooled analysis. The number of nematode eggs produced differed among genotypes, with the greatest number of eggs (numerically) produced on Dale and the fewest on GT-IR7-BC1F6 (Table 5). The recurrent parental genotypes (Dale, Top 76-6, Collier, GT-IR7, and Entry 22) were all statistically similar to each other, and the BC_1_F_6_ genotypes (Dale-BC1F6, Top 76-6-BC1F6, Collier-BC1F6, GT-IR7-BC1F6, and Entry 22-BC1F6) were similar to each other and to the resistant standard, Honey Drip (Table 5).

**Table 5.** Total *M. incognita* eggs and eggs/gram of root of parental sorghum genotypes and their BC_1_F_6_ progeny that have the *M. incognita* resistance QTL QTL-Sb.RKN.3.1.

Genotype	Total eggs^a^		Eggs/g root^a^	
Dale	425775	A^b^	11141	A^b^
Top 76-6	297250	A	3977	A
Collier	206325	A	5856	A
GT-IR7	170125	A	5584	A
Entry 22	115808	A	4661	A
Honey Drip	2300	B	48	B
Collier-BC1F6	1175	B	36	B
Entry 22-BC1F6	975	B	20	B
Dale-BC1F6	875	B	19	B
Top 76-6-BC1F6	750	B	18	B
GT-IR7-BC1F6	625	B	16	B

Notes: ^a^Combined data from two trials. Data was collected 8 weeks after infection with *M. incognita.* Statistical analysis performed using log_10_ transformed data, however, untransformed numbers are presented in the table.

b Means within a column followed by the same letter are not statistically different (*P* ≤ 0.05).

Because root weights differed among genotypes, nematode reproduction per gram of root was also calculated. Although the mean number of eggs per gram of root changed the numerical ranking of the genotypes, the results were similar to those for the total number of eggs produced. The recurrent parental genotypes were all statistically similar to each other, and the BC_1_F_6_ genotypes were similar to each other and to the resistant standard, Honey Drip (Table 5).

## Discussion

Host-plant resistance to a nematode species is a relative term that is based on comparing the level of nematode reproduction on a plant genotype to the level of reproduction on a designated susceptible genotype of the same species. A genotype that reduces reproduction by 90% is typically acknowledged as highly resistant, whereas smaller reductions are often called partially or moderately resistant (Davis and Stetina, 2016; Hussey and Janssen, 2002). Levels of *Meloidogyne* spp. reproduction can be documented in two different ways: total number of eggs produced and eggs per gram of root produced. Standardizing the amount of reproduction on a per gram of root basis is often used when the plants being evaluated have large differences in root mass. However, the two measurements address different questions. The total reproduction is useful for evaluating whether a genotype is likely to suppress nematode levels in a field and therefore be beneficial to a subsequent susceptible crop. Reproduction per gram of root is useful for evaluating the parasitic load on that plant (Davis and Stetina, 2016), which should be correlated with the amount of damage caused to that crop. In the study reported herein, all BC_1_F_6_ genotypes would be considered highly resistant to *M. incognita* based on either total reproduction or reproduction per gram of root. Therefore, we conclude that all of the BC_1_F_6_ genotypes should suffer little or no damage from *M. incognita* and also be effective at suppressing *M. incognita* in the field thereby serving as an effective rotation crop.

Movement of favorable alleles at QTL regions into different plant backgrounds is an important next step after the identification of QTL for a trait. Although a QTL may account for a large amount of the phenotypic variance of a trait, the movement of favorable alleles in this QTL region into a different genetic background may not always confer the desired trait to the progeny. For example, a single recessive gene conferring resistance to the *Zucchini yellow mosaic virus* Florida strain (ZYMV-FL) was identified in a watermelon (*Citrullus lanatus*) F_2_ mapping population of PI 595203 (resistant) x ‘New Hampshire Midget’ (NHM, susceptible) (Ling et al., 2009). When the QTL region was moved using marker-assisted selection from PI 595203 into the ‘Charleston Gray’ background, the resulting BC_2_F_2_ plants did not exhibit the same level of resistance found in PI 595203 as they exhibited virus symptoms and virus replication was detected on even the most resistant plants (Harris et al., 2009). The authors speculated that a modifier gene controlling ZYMV-FL replication may exist in ‘Charleston Gray’.

Marker-assisted selection has been used to move disease resistance QTL from a donor parent to a recurrent parent resulting in resistant phenotypes. Resistance to rice (*Oryza sativa*) blast, caused by *Magnaporthe oryzae*, and sheath blight, caused by *Rhizoctonia solani*, was moved from the donor line Tetep to the susceptible rice hybrid Pusa 6B (Singh et al., 2015). Blast resistance is controlled by the blast resistance gene *Pi54* and sheath blight resistance is controlled by three QTL. BC_1_F_2_ plants that were homozygous for *Pi54* were selfed to generate BC_1_F_3_ families that were then subjected to a stepwise reductive screening utilizing markers for the three sheath blight resistance QTL. The BC_1_F_5_ plants were phenotyped and the lines containing the blast and sheath blight resistance QTL in the Pusa 6B background were resistant to both rice blast and sheath blight.

Following the original cross between the susceptible sorghum line Collier and the resistant line Honey Drip, F_1_ plants were found to be resistant to *M. incognita*, and the F_2_ generation segregated in approximately a 3:1 ratio of resistant to susceptible (Harris-Shultz et al., 2015a). Thus, the resistance was inherited as a single dominant locus. In the study reported herein, incorporating QTL-Sb.RKN.3.1 into a diverse set of genotypes resulted in a high level of resistance in all genotypes, which suggests that the resistance QTL is likely to confer resistance to *M. incognita* when incorporated into other genetic backgrounds. If any of the BC_1_F_6_ genotypes evaluated for this study were crossed to a susceptible line, it is expected that the resulting F_1_ hybrid would be highly resistant to *M. incognita*.

The effectiveness of using crop rotation to minimize damage from *Meloidogyne* spp. to a crop of primary economic importance has been known for more than a century (Bessey, 1911). To effectively manage nematode populations in a cropping system, each crop should leave a lower nematode population density than the economic damage threshold of the following crop (Nusbaum and Ferris, 1973). Cotton is one of the primary field crops in the southern United States, and although several species of nematodes can cause severe damage to cotton, *M. incognita* causes the greatest total loss because of its widespread distribution (Davis and Stetina, 2016; Davis et al., 2018). Additional options for rotation crops that are profitable and do not require expensive, specialized equipment would be beneficial, and *M. incognita*-resistant crops such as sorghum would increase the options available for nematode management in cotton. Additionally, *Meloidogyne*-resistant crops can provide benefits beyond suppression of the target nematode species. For example, in contrast to the effect of some nematicides (Timper et al., 2012), host resistance to a plant-parasitic nematode should not affect population levels of beneficial nematodes or most other soil organisms, which may allow fields to maintain a level of natural suppression of nematodes or other pathogens.

In this study we moved the resistance QTL from ‘Honey Drip’ into five different sorghum backgrounds that included forage, sweet, and grain sorghum. The resistance to *M. incognita* in all of the backcross lines was equivalent to ‘Honey Drip’ and suggests this gene could be introgressed using marker-assisted selection into many sorghum genotypes and confer resistance. Thus, this QTL and its associated markers will be useful for sorghum breeding programs that want to incorporate *M. incognita* resistance into their sorghum lines.
